# Biased Safety Reporting in Blinded Randomized Clinical Trials: Meta-Analysis of Angiotensin Receptor Blocker Trials

**DOI:** 10.1371/journal.pone.0075027

**Published:** 2013-09-23

**Authors:** Nobuyoshi Takabayashi, Hisashi Urushihara, Koji Kawakami

**Affiliations:** Department of Pharmacoepidemiology, Graduate School of Medicine and Public Health, Kyoto University, Kyoto, Japan; Osaka University Graduate School of Medicine, Japan

## Abstract

**Background:**

Cough is listed as an adverse drug reaction (ADR) on the labels of angiotensin receptor blockers (ARB). However, a causal association with cough has also been reported for angiotensin converting enzyme inhibitors (ACEI), which have frequently been used as comparator drugs in the registration clinical trials of ARBs. This prompted us to examine the possible influence of using comparator drugs with well-known ADRs on the safety reporting of investigational drugs in blinded randomized clinical trials.

**Methods and Findings:**

The double-blinded, randomized clinical trials with comparator drugs were identified in the Japanese dossiers for the new drug applications of ARBs. The risk ratios (RR) of reporting cough and headache in ARB arms were calculated for each ARB by comparing trials using ACEIs and trials using non-ACEIs, were then combined with a meta-analysis. 23 trials with a total of 6643 patients were identified, consisting 6 trials using an ACEI comparator including 819 ARB patients and 17 trials using a non-ACEI comparator including 5824 ARB patients. The combined RR of cough reporting was significantly elevated (20.77; 95% confidence interval [CI], 7.47 to 57.76), indicating more frequent reporting of cough in clinical trials using an ACEI comparator. In contrast, the combined RR of headache, a negative control, was insignificant (1.45; 95% CI, 0.34 to 6.22).

**Conclusion:**

The use of comparators with well-known ADRs in blinded randomized trials produces potential bias in the reporting frequency of ADRs for investigational drugs. The selection of appropriate comparator drugs should be critical in unbiased safety assessment in double-blinded, randomized clinical trials and thus have relevance in reviewing the safety results from a regulatory point of view.

## Introduction

The association of angiotensin converting enzyme inhibitors (ACEI) with cough has long been known, and involves the accumulation of bradykinin through an inhibitory effect on angiotensin converting enzyme [1], [2]. Japanese Package Inserts and US labels also frequently list cough as an adverse drug reaction (ADR) of angiotensin receptor blockers (ARB), despite the lack of evidence supporting a causal pharmacological relationship with this symptom.

ACEIs are frequently used as comparator drugs in clinical trials of other classes of antihypertensives in both development and post-marketing phases, including double-blinded, randomized controlled phase III clinical trials of ARBs for marketing applications [3]–[8]. Recognition by investigators that ACEI is a comparator drug in the clinical trials of an ARB likely results in the expectation of an elevated incidence of cough in the ACEI treatment arm, and might result in a high reporting frequency of cough due to heightened attention to it not only in the blinded ACEI arm in such trials but also in the blinded investigational ARB arm.

Randomization and treatment allocation masking in clinical trials are established standards in minimizing selection bias, information bias, and confounding, and thereby help ensure the validity of the results [9]. However, such study design may carry the risk of introducing reporting bias arising from the use of a comparator drug with known adverse reactions. To our knowledge, the bias caused by the use of comparator drugs in reporting ADR/adverse events (AEs) in randomized, double-blinded clinical trials has not been investigated.

To investigate the effect of comparator drugs with well-known ADRs on the safety reporting of investigational drugs, we compared the reporting frequencies of cough in the clinical trials of ARB which used enalapril as comparator drug with those for the same ARB in trials using a non-ACEI drug as comparator.

## Methods

### Design

Our working hypothesis was that the frequency of cough reported for a certain ARB in clinical trials which used an ACEI drug as a comparator drug (hereinafter referred to as ‘ACEI trials’) would be higher than that in clinical trials of the same ARB agent which used a non-ACEI comparator (non-ACEI trials).

To test this hypothesis, we pooled the incidence of cough reported as an AE (adverse event regardless of causal relationship with the drug concerned) or ADR (adverse drug reaction: AE for which an investigator indicates a causal association with the drug concerned) in ARB arms across non-ACEI trials and compared it with that for the same ARB in clinical trials using enalapril as comparator. We investigated the results of clinical trials for new drug application (NDA) in Japan because these trials were required to meet uniform compliance with regulations and GCP standards in a single country, which in turn ensures data quality for submission of the trial results and complete data availability through mandatory information disclosure. Further, for each ARB agent investigated in this study, the same sponsors conducted all registration trials for NDA throughout the development program of that agent, and we expected that the in-house standard operation procedures for conducting clinical trials would be consistent within the same ARB sponsors. We therefore considered that pooled analysis of multiple trials of the same ARB agent was justifiable for comparison of cough incidence between ACEI and non-ACEI trials.

Additionally, we used headache as a negative control event, because we expected that having ACEI as comparator drug would have no effect on the reporting of headache for ARB agents. In fact, headache is common in patients with hypertension and is listed in the package inserts of many antihypertensives, including ARB agents.

### Selection and search of trials

The study covered the trials of six ARBs, namely losartan, candesartan, valsartan, olmesartan, telmisartan and irbesartan, on the basis of their having been marketed in Japan as of Nov 2011. The package inserts for these ARBs all list cough as an ADR.

Eligible trials met all of the following criteria: 1) registered clinical trial of an ARB for NDA whose results were available through the application dossier; 2) randomized control, double-blind design; and 3) conducted in Japan with Japanese patients with essential hypertension before the first market launch. We focused on Japanese patients to minimize intrinsic and extrinsic effects, given that the incidence of cough is subject to racial differences and may be affected by other factors, such as lifestyle [10], [11]. Moreover, conduct in a single country ensures the uniformity of data quality, and precludes the impact of environmental factors such as medical care systems and regulations for clinical trials. Short-term trials were included on the basis that the occurrence of cough with ACEI has been reported as early as within 4 weeks after the start of treatment [12].

Application dossiers of ARBs for NDA submitted from Sep 1999 to Nov 2011 were obtained by searching of the publicly available Pharmaceuticals and Medical Devices Agency (PMDA) website, and by disclosure requests for the dossier of losartan to the Ministry of Health, Labor and Welfare and for that of candesartan to Takeda Pharmaceutical Co., Ltd [13]. In addition, we searched academic publications on the eligible trials identified in the application dossiers of ARBs via PubMED for the English literature and I-Chu-Shi web for Japanese publications.

Searches were executed according to the study protocol, and the last search was run on 24 Jan 2012. This review was reported in accordance with the PRISMA guidelines (Checklist S1) [14].

### Data extraction and collection

Two independent reviewers (N. T. and H. U.) reviewed the submission dossiers for marketing registration and identified eligible trials. Risk of bias in eligible trials was assessed using the Cochrane Collaboration tool for result synthesis [15]. Discrepancies in trial selection and evaluation of the risk of bias between reviewers were resolved by discussion. The incidence of ADRs and AEs, including cough and headache, reported in the eligible trials, as well as trial design, trial duration, comparator drug, patient age and sex were obtained (Table S1).

### Statistical analysis

The number of ADRs and AEs was counted by the patient unit. Risk ratios (RR) representing the degree of ‘bias’ in the reporting of cough and headache in each ARB arm were calculated for ADRs and AEs separately, as follows. Reporting frequency was defined as the number of events divided by the total number of patients in the group. The ‘biased’ reporting frequency of an event derived from the ARB arm of an ACEI trial was divided by the ‘non-biased’ reporting frequency of the event, which was the pooled number of incidences of ADRs and AEs for the event divided by the total number of patients in the same ARB arms across all eligible non-ACEI trials. We combined these RRs across all ARB agents by meta-analysis to yield a single common estimate. A lower limit of the 95% confidence interval of the combined RR above 1.0 would suggest potential bias in reporting due to awareness of the safety profile of the comparator drug. To calculate the combined RR, in case that zero events were reported in any single ARB arm of an ACEI trial or in any pooled ARB arm across non-ACEI trials for a single ARB agent (i.e. zero cell), a half integer was added to each zero cell according to the classic statistical correction method. Meta-analysis was conducted using the random effects model of DerSimonian and Laird, because we combined the clinical trials of different ARB agents [16]. Heterogeneity was shown by the I-squared statistic (%) and p-value [17], [18]. Sensitivity analysis was conducted by omitting the most influential trial from meta-analysis.

Proportional reporting ratio (PRR) is a commonly used index in post-marketing safety surveillance to detect safety signals [19]. In this study, PRR was defined as the ratio of the proportion of cough reported in total ADR reports pooled for all ARB agents in ACEI trials to that in non-ACEI trials, and thereby represented the degree of biased reporting of cough for ABRs in ACEI trials. We made the same calculations for AE reporting and for headache.

All analyses were conducted with STATA version 12.0 (STATA Corp).

## Results

**Figure 1:**
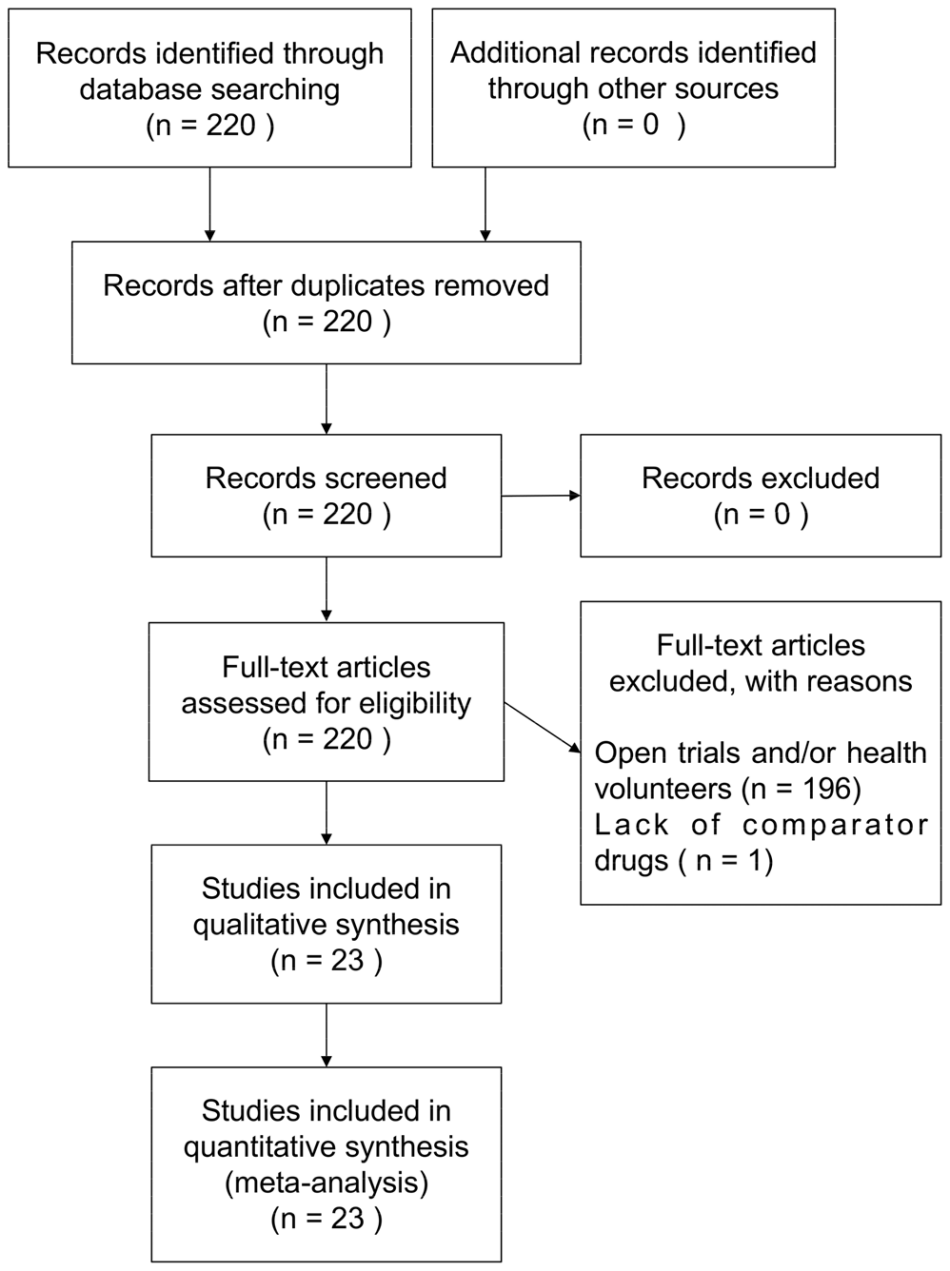
Flow diagram for stydy inclusion and exclusion.

Of 220 trials in the submission dossiers of 6 ARB agents, 23 were identified as eligible for the analysis of ADR reporting (Table S1) [3]–[8], [20], [21]. 197 trials were excluded because of an ineligible trial population, such as healthy volunteers, or an ineligible study design, such as open-label or lack of comparator drugs (Figure 1). Only one trial using enalapril as a comparator was conducted for each ARB agent. A total of 6643 patients in the 23 trials consisted of 819 patients in the ARB arms of 6 ACEI trials and 5824 patients in the ARB arms of 17 non-ACEI trials. Mean patient age ranged from 56 to 57 years in the ACEI trials, and from 51 to 60 years in the non-ACEI trials, while trial duration ranged from 1 to 12 weeks. AE incidences were derived from only 16 trials; 1 losartan trial and 1 valsartan trial with an ACEI agent as comparator did not report AE incidence in their clinical summary reports and were excluded from analysis in AE reporting. In AE analysis, 3732 patients in the 16 trials consisted of 523 patients in the ARB arms of 4 ACEI trials and 3209 patients in the ARB arms of 12 non-ACEI trials. The characteristics of trials included for AE reporting were similar to those for ADR reporting.

Although relatively few episodes of cough were reported as an ADR for ARB agents, frequencies in ACEI trials were nevertheless much higher than in non-ACEI trials (Table 1). This tendency was also seen for AE reporting. With regard to headache, in contrast, ADR reporting frequency in ACEI trials was similar to that in non-ACEI trials (Table 2).

**Table 1:** Cough incidence in clinical trials of angiotensin receptor blockers by comparator drugs.

	vs. ACEI				vs. non-ACEI			
ARB class	N of trials	N of patients	ADR	AE	N of trials	N of patients	ADR	AE
Losartan	1	144	1	NA	3	838	1	NA
Candesartan	1	134	2	2	3	1026	1	3
Valsartan	1	152	4	NA	3	1777	1	NA
Olmesartan	1	148	2	5	3	760	0	7
Telmisartan	1	106	7	11	4	1186	0	4
Irbesartan	1	135	4	12^a^	2	237	1	1
Total	6	819	20	30	17^b^	5824	4	15

a Part of the data include number of events, instead of number of patients.

b One trial included one Losartan arm and one Irbesartan arm (Yoshinaga, 2008 [21]).

ARB, angiotensin receptor blocker; ACEI, angiotensin converting enzyme inhibitor; ADR, adverse drug reaction; AE, adverse event; NA, not applicable.

**Table 2:** Headache incidence in clinical trials of angiotensin receptor blockers by comparator drugs.

	vs. ACEI				vs. non-ACEI			
ARB class	N of trials	N of patients	ADR	AE	N of trials	N of patients	ADR	AE
Losartan	1	144	1	NA	3	838	6	NA
Candesartan	1	134	0	0	3	1026	2	17^a^
Valsartan	1	152	0	NA	3	1777	13	NA
Olmesartan	1	148	0	6	3	760	7	23
Telmisartan	1	106	3	13	4	1186	0	16
Irbesartan	1	135	1	10^a^	2	237	2	12
Total	6	819	5	29	17^b^	5824	30	68

a Part of the data include number of events, instead of number of patients.

b One trial included one Losartan arm and one Irbesartan arm (Yoshinaga, 2008 [21]).

ARB, angiotensin receptor blocker; ACEI, angiotensin converting enzyme inhibitor; ADR, adverse drug reaction; AE, adverse event; NA, not applicable.

Among the 23 eligible trials, no study was assessed to be at high risk of bias according to the Cochrane tool, and no trial was accordingly excluded from subsequent meta-analysis (Table S2). The RRs for ‘biased’ ADR reporting of cough were significant for all ARBs except for losartan and irbesartan, which had insignificant elevations. Of note, the highest RR (and wide confidence interval) of ADR reporting of cough was found for telmisartan.

**Figure 2:**
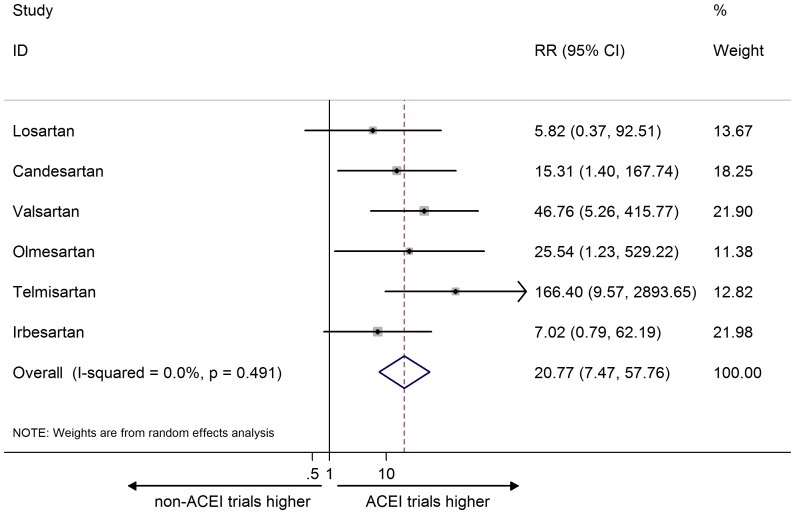
Meta-analysis of risk ratio of the adverse drug reaction for cough. Error bars indicate 95% CIs; ARB, angiotensin receptor blocker; ACEI, angiotensin converting enzyme inhibitor; RR, risk ratio. Diamonds represent pooled RR with 95% CI.

**Figure 3:**
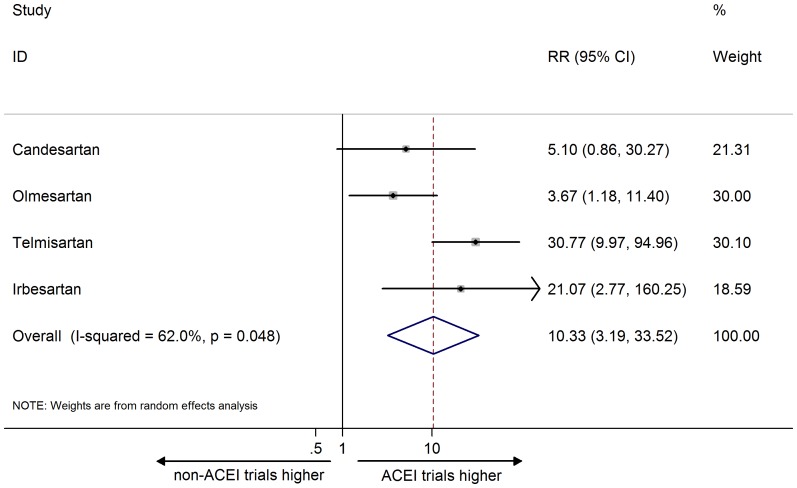
Meta-analysis of risk ratio of the adverse event for cough. Error bars indicate 95% CIs; ARB, angiotensin receptor blocker; ACEI, angiotensin converting enzyme inhibitor; RR, risk ratio. Diamonds represent pooled RR with 95% CI.

The combined RR of cough ADR reporting across six ARB agents was statistically significant, indicating that cough ADR reporting for ARB was more frequent in ACEI than in non-ACEI trials (20.77, 95% CI 7.47 to 57.76; I^2^ = 0%; Figure 2). The same observation was repeated for the combined RR for AE reporting of cough (10.33, 95% CI 3.19 to 33.52; I^2^ = 62.0%; Figure 3).

**Figure 4:**
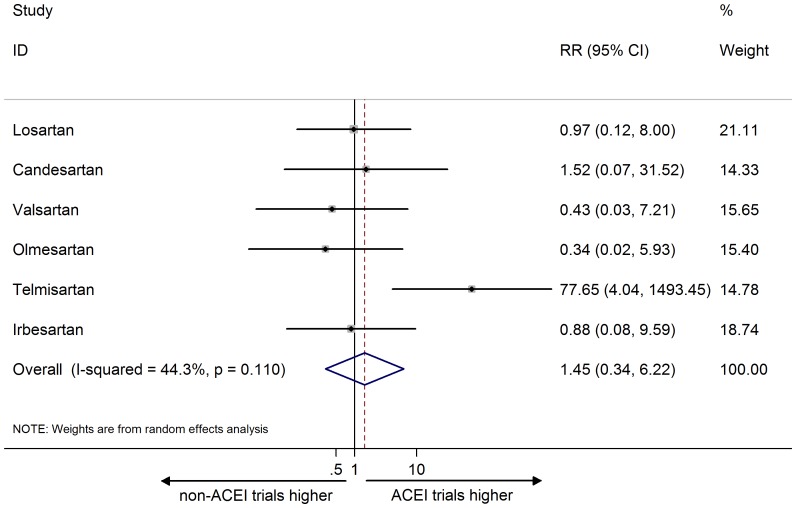
Meta-analysis of risk ratio of the adverse drug reaction for headache. Error bars indicate 95% CIs; ARB, angiotensin receptor blocker; ACEI, angiotensin converting enzyme inhibitor; RR, risk ratio. Diamonds represent pooled RR with 95% CI.

**Figure 5:**
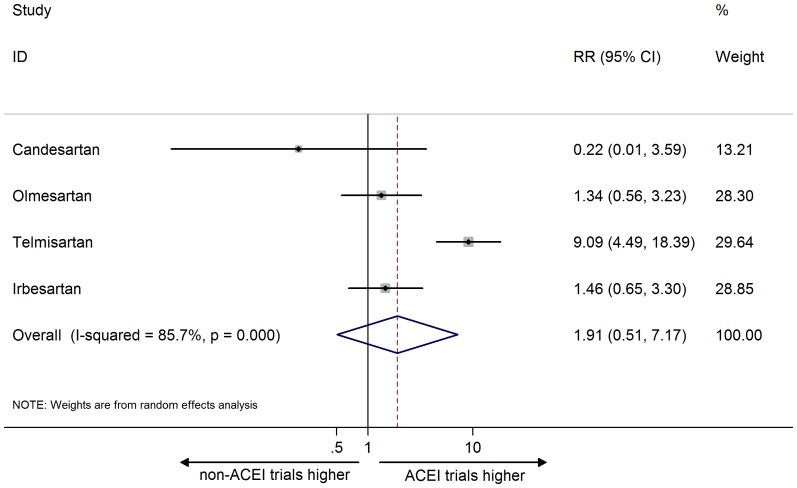
Meta- analysis of risk ratio of the adverse event for headache. Error bars indicate 95% CIs; ARB, angiotensin receptor blocker; ACEI, angiotensin converting enzyme inhibitor; RR, risk ratio. Diamonds represent pooled RR with 95% CI.

In contrast, the combined RR for headache ADR reporting was not significant, indicating that headache reporting between ACEI and non-ACEI trials was similar (1.45, 95% CI 0.34 to 6.22; I^2 = ^44.3%; Figure 4). The combined RR for headache AE reporting was also insignificant (RR: 1.91, 95% CI 0.51 to 7.17; I^2 = ^85.7%; Figure 5).

PRR for ADR and AE reporting of cough indicated the same tendency as seen in the meta-analysis of significantly higher reporting of cough in ACEI trials than non-ACEI trials (for ADR reporting: 24.68, 95%CI 8.54 to 71.28; for AE reporting: 7.90, 95%CI 4.29 to 14.52). Further, PRR for ADR and AE reporting of headache also showed similar results to the above meta-analysis (for ADR reporting: 0.82, 95%CI 0.32 to 2.09; for AE reporting: 1.68, 95%CI 1.11 to 2.56).

In sensitivity analyses, exclusion of the highest RRs, for telmisartan, did not alter the significance of results for combined RRs (for ADR reporting: 15.30, 95% CI 5.12 to 45.74; I^2 = ^0%; for AE reporting: 5.88, 2.08 to 16.65; 24.2%). For headache, moreover, the combined RRs after excluding telmisartan studies remained statistically insignificant for both ADR (0.75, 0.24 to 2.35; 0%) and AE reporting (1.30, 0.72 to 2.32; 0%).

## Discussion

In this study, we examined whether the use of comparator drugs with well-known ADRs can bias ADR reporting by investigators for investigational drugs in double-blinded, randomized clinical trials. For ADR reporting, the relative reporting frequency of cough was significantly higher in the ARB arms of ACEI trials than that in non-ACEI trials. This tendency was repeated for AE reporting. In contrast, no elevation in reporting frequency was observed in the ARB arms of ACEI trials for headache. These observations support the existence of a comparator effect in safety reporting for investigational drugs in the setting of double-blinded, randomized trials.

To our knowledge, this is the first report to suggest a possible information bias related to a comparator drug in safety reporting under a randomized and double-blinded design. Investigators’ knowledge on an association of ACEI agents with cough would likely have prejudiced them to pay attention to and report cough more frequently, even in the blinded ARB arms of ACEI trials. The mechanism of this ‘comparator effect’ seems similar to the ‘notoriety bias’ seen in post-marketing spontaneous reporting, in which the number of reports is inflated due to heightened attention to a particular ADR [22], [23].

ADR reporting appears to be more prone to this type of bias than AE reporting, since ADRs are reported after determining causality of an individual adverse event with a suspected drug, a process which is heavily dependent on the personal judgment of the reporting investigator. This may explain why the combined RRs for cough ADR reporting in the present study were larger than those for AE reporting.

Given that the association of cough with enalapril first came to attention following the 1985 report [1], the reported incidence of cough associated with enalapril should likely differ before and after this knowledge was obtained. Before the 1986 marketing approval in Japan, the reported incidence of cough for enalapril was as low as 0.44% (4/912) [24]. In contrast, higher ADR incidences of cough were reported for enalapril in clinical trials of ARBs conducted from 1993 to 2001 (13.3% vs. losartan to 26.5% vs. olmesartan). Further, the US Physicians’ Desk Reference lists an ADR incidence of cough for enalapril of 1.3%, a result which has not been updated since 1991 at least. In contrast, a cough incidence of 11.48% was reported for enalapril-related ADR in a meta-analysis of the literature [25]. These findings also support the notion that a greater awareness of a causal association with a particular ADR causes its more frequent reporting.

This comparator effect on safety reporting in Japan might be supported by observations in the clinical trials of celecoxib in patients with osteoarthritis [26]. The reported frequency of gastrointestinal ADRs for celecoxib was as low as 5.1% (6/117) in the placebo-controlled phase II trials despite a frequency of 19.4% (73/377) in the phase III trials with an NSAID, loxoprofen, as comparator drug, which is a well-known cause of gastrointestinal ADRs.

Excess safety reporting by comparator effects in phase III trials would result in increased frequencies of ADRs described in package inserts; the safety results derived from phase III trials, which usually use active comparators, generally dominate the safety profile of a newly approved chemical entity. Regulatory reviewers and sponsor companies should be aware of this potential bias when reviewing safety results from randomized, blinded trials with active comparators with well-known adverse reactions.

With regard to methodology, we considered that pooling and comparing frequencies in the treatment arms of individual ARBs across trials conducted by the same sponsor companies was valid in yielding an RR, for the following reasons: the trial population and design, such as with regard to the disease definition of essential hypertension and eligibility criteria, including outpatients and prohibited concomitant therapies, were basically consistent during development of an individual ARB agent as a result of sponsor adherence to guidelines and principles for the clinical evaluation for development of antihypertensive drugs [27], [28]. Patient characteristics were sufficiently alike to allow comparison within clinical trials for the individual ARB agent. Further, the quality assurance system employed for registration trials was consistent within the same sponsor companies for the sole common purpose of Japanese regulatory approval.

We consider that our results are free of publication and language bias because all the clinical trials sponsored by the developing companies were required to be registered at the regulatory agency, and were then listed and available in the application dossiers for marketing registration, even if these subsequently failed.

Further, the insignificant effect of comparator on the reporting frequency of headache as a negative control suggests that the observed significance in cough reporting was not a result of chance. The results of PRR analysis for ADR and AE reporting were consistent with the results of meta-analysis. The significantly elevated PRR of AE reporting for headache was small and seemed negligible in practice. Sensitivity analysis after the exclusion of RRs for telmisartan trials remained essentially unchanged, confirming the robustness of our results. The telmisartan trial using enalapril as a comparator drug was conducted immediately after the introduction of new GCP guidelines in April, 1997 in Japan [29]. At that time, clinical trial data were collected with rigorous source data verification against medical charts, probably leading to temporarily higher overall AE reporting in this trial [7].

Several limitations warrant mention. As reported in trials with cardiovascular endpoints, consideration should be given to differences in coding procedures and dictionary of AE among sponsoring companies when RRs for each ARB agent are combined [30]. In our study, however, sponsor-specific aspects in reporting and summarizing AEs were likely cancelled when RRs were calculated by comparing reporting frequencies for the single ARB agent developed by same sponsor companies, as discussed above. We therefore consider that the impact of any differences in coding definitions and procedures would be minimal. The half-integer correction was applied to zero observations found in reporting of cough and headache in several trials to calculate the combined RRs. This correction, in the case of cough, has a potential to underestimate the combined RR. However, this potential underestimation would not influence our conclusion since the RR value after the correction was significant. In addition, the PRR analysis, to which zero event correction is inapplicable, supported the results of the meta-analysis. For an individual ARB agent, only a single ACEI trial was available for analysis and therefore the total number of patients receiving a certain ARB in the ACEI trial was imbalanced with that in multiple non-ACEI trials. However, this imbalance is unlikely to have produced crucial bias in the calculation of combined RRs for ADR and AE reporting, given that all the calculated RRs for each ARB were in the same direction.

In conclusion, our findings suggest that the effect of a comparator with well-known ADRs in a blinded randomized trial produces potential bias in the reporting frequency of ADRs for an investigational drug. The selection of appropriate comparator drugs is critically important in double-blinded, randomized clinical trials, particularly for new chemical entities under development, and thus regulators, health-care practitioners and sponsor companies should be aware of its relevance in the safety evaluation.

## Supporting Information

Table S1 Characteristics of eligible trials.
(DOC)

Table S2 Evaluation of risk of bias.
(DOC)

Checklist S1 **PRISMA checklist.**
(DOC)
