# Supplementary material for: Biased Safety Reporting in Blinded Randomized Clinical Trials: Meta-Analysis of Angiotensin Receptor Blocker Trials
Source: PLoS One. 2013 Sep 23;8(9):e75027. doi: 10.1371/journal.pone.0075027 (PMC3781030; doi:10.1371/journal.pone.0075027)
Supplement: Table S1 — Characteristics of eligible trials. (DOC) [file pone.0075027.s001.doc]

Table S1. Characteristics of eligible trials.

| Study | ARB | Subject population | Patient status | Duration | Dose schedule | Comparator drug | Arm | Comparator type | No. of patients | Age, y  mean (SD) | Men, % |
| --- | --- | --- | --- | --- | --- | --- | --- | --- | --- | --- | --- |
| Yoshinaga et al, 1995 [3] | L | Mild-moderate essential HT | Out | 12w | Escalation | Enalapril | L25-50 | ACEI | 144 | 56(10) | 36.1 |
| Sumita et al, 1995 [20] | L | Mild-moderate essential HT | Out/In | 1w | Fixed | Placebo | L50 | non-ACEI | 8 | 58.1(6.8) | 25.0 |
| No 301 | L | Essential HT | Out | 8w | Fixed | HCTZ, Placebo | L50/H12.5 | non-ACEI | 155 | 54.7(9.9) | 57.1 |
|  |  |  |  |  |  |  | L50/H6.25 | non-ACEI | 162 | 56.1(8.9) | 64.2 |
|  |  |  |  |  |  |  | L25/H6.25 | non-ACEI | 155 | 54.9(10.3) | 59.5 |
|  |  |  |  |  |  |  | L50 | non-ACEI | 160 | 55.5(9.9) | 61.8 |
| Yoshinaga, 2008 [21] | L | Mild-moderate essential HT | Out | 8w | Escalation | Irbesartan | L50-100 | non-ACEI | 198 | 58(28-79)^a^ | 76.1 |
| Arakawa et al, 1998 [4] | C | Mild-moderate essential HT | Out | 12w | Escalation | Enalapril | C4-8-12 | ACEI | 134 | 56.4(9.9) | 38.1 |
| CCT-002 | C | Mild-moderate essential HT | Out | 8w | Fixed | HCTZ | C4/H6.25 | non-ACEI | 148 | 54.1(10.7) | 64.9 |
|  |  |  |  |  |  | HCTZ | C8/H6.25 | non-ACEI | 148 | 53.6(10.5) | 63.5 |
|  |  |  |  |  |  |  | C8 | non-ACEI | 148 | 54.8(10.3) | 58.1 |
| CCT-001 | C | Mild-moderate essential HT | Out | 8w | Fixed | HCTZ, Placebo | C4 | non-ACEI | 69 | 54.8(12.0) | 63.8 |
|  |  |  |  |  |  |  | C8 | non-ACEI | 68 | 53.0(10.1) | 70.6 |
|  |  |  |  |  |  |  | C4/H12.5 | non-ACEI | 69 | 54.4(9.7) | 59.4 |
|  |  |  |  |  |  |  | C8/H12.5 | non-ACEI | 68 | 56.4(9.1) | 60.3 |
| CCT-001 | C | Mild-moderate essential HT | Out | 12w | Fixed | Amlodipine, Placebo | C8/A5 | non-ACEI | 101 | 56.6(10.8) | 59.4 |
|  |  |  |  |  |  |  | C8/A2.5 | non-ACEI | 36 | 57.4(11.5) | 58.3 |
|  |  |  |  |  |  |  | C8 | non-ACEI | 100 | 56.9(11.3) | 67.0 |
|  |  |  |  |  |  |  | C4/A5 | non-ACEI | 36 | 56.9(8.3) | 44.4 |
|  |  |  |  |  |  |  | C4/A2.5 | non-ACEI | 35 | 55.9(9.9) | 54.3 |
| Yoshinaga et al, 1998 [5] | V | Essential HT | Out | 12W | Escalation | Enalapril | V40-80-160 | ACEI | 152 | 55.9(10.2) | 48.0 |
| 1301 | V | Mild-moderate essential HT | Out | 8w | Fixed | HCTZ, Placebo | V80/H12.5 | non-ACEI | 103 | 52.8(9.9) | 67.0 |
|  |  |  |  |  |  |  | V40/H12.5 | non-ACEI | 102 | 54.6(9.9) | 68.6 |
|  |  |  |  |  |  |  | V80 | non-ACEI | 101 | 54.9(8.6) | 73.3 |
|  |  |  |  |  |  |  | V40 | non-ACEI | 102 | 53.4(9.6) | 71.6 |
| 1303 | V | Mild-moderate essential HT | Out | 8w | Fixed | HCTZ, Placebo | V80/H12.5 | non-ACEI | 66 | 52.8(10.5) | 65.2 |
|  |  |  |  |  |  |  | V80/H6.25 | non-ACEI | 64 | 54.7(11.5) | 67.2 |
|  |  |  |  |  |  |  | V40/H12.5 | non-ACEI | 65 | 54.9(9.3) | 63.1 |
|  |  |  |  |  |  |  | V40/H6.25 | non-ACEI | 61 | 54.7(8.3) | 63.9 |
|  |  |  |  |  |  |  | V80 | non-ACEI | 68 | 53.3(11.2) | 61.8 |
|  |  |  |  |  |  |  | V40 | non-ACEI | 65 | 54.8(10.1) | 56.9 |
| 1301 | V | Mild-moderate essential HT | Out | 8w | Fixed | Amlodipine, Placebo | V40/A2.5 | non-ACEI | 159 | 53.9(10.3) | 71.1 |
|  |  |  |  |  |  |  | V40/A5 | non-ACEI | 167 | 52.8(10.7) | 70.7 |
|  |  |  |  |  |  |  | V80/A2.5 | non-ACEI | 160 | 52.8(10.8) | 75.0 |
|  |  |  |  |  |  |  | V80/A5 | non-ACEI | 162 | 54.1(9.8) | 75.9 |
|  |  |  |  |  |  |  | V40 | non-ACEI | 169 | 52.9(10.1) | 77.5 |
|  |  |  |  |  |  |  | V80 | non-ACEI | 163 | 52.6(11.3) | 69.9 |
| Arakawa et al, 2004 [6] | O | Mild-moderate essential HT | Out | 12w | Escalation | Enalapril | O10-20-40 | ACEI | 148 | 56.8(9.7) | 48.9 |
| 5-34 | O | Mild-moderate essential HT | Out | 2w | Fixed | Placebo | O10 | non-ACEI | 20 | 56.5(6.8) | 45.0 |
|  |  |  |  |  |  |  | O20 | non-ACEI | 19 | 50.8(8.3) | 73.7 |
| J301 | O | Mild-moderate essential HT | Out | 12w | Fixed | Azelnidipine | O20AZ16 | non-ACEI | 215 | 56.6(10.5) | 68.4 |
|  |  |  |  |  |  |  | O10/AZ8 | non-ACEI | 221 |
|  |  |  |  |  |  |  | O20 | non-ACEI | 213 |
| J201 | O | Mild-moderate essential HT | Out | 4w | Fixed | Azelnidipine | O10/AZ8 | non-ACEI | 36 | 57.9(97) | 55.0 |
|  |  |  |  |  |  |  | O10 | non-ACEI | 36 |
| Arakawa et al, 2002 [7] | T | Mild-moderate essential HT | Out | 12w | Escalation | Enalapril | T20-40-80 | ACEI | 106 | 57(10) | 64.4 |
| 502.439 | T | Mild-moderate essential HT | Out | 8w | Fixed | HCTZ, Placebo | T40 | non-ACEI | 63 | 55.5(10.0) | 55.6 |
|  |  |  |  |  |  |  | T40/H12.5 | non-ACEI | 64 | 55.5(9.5) | 68.8 |
|  |  |  |  |  |  |  | T80 | non-ACEI | 66 | 53.5(9.7) | 51.5 |
|  |  |  |  |  |  |  | T80/H12.5 | non-ACEI | 64 | 54.6(10.9) | 70.3 |
|  |  |  |  |  |  |  | T40/H6.25 | non-ACEI | 62 | 55.7(10.2) | 54.8 |
|  |  |  |  |  |  |  | T80/H6.25 | non-ACEI | 66 | 54.2(7.7) | 65.2 |
| 502.436 | T | Mild-moderate essential HT | Out | 8w | Fixed | HCTZ | T40 | non-ACEI | 109 | 56.1(7.9) | 60.6 |
|  |  |  |  |  |  |  | T40/H12.5 | non-ACEI | 109 | 56.6(9.6) | 52.3 |
| 1235.13 | T | Essential HT | Out | 8w | Escalation | Amlodipine | T20/A5-T40/A5 | non-ACEI | 269 | 57.0(9.6) | 73.2 |
| 1235.14 | T | Essential HT | Out | 8w | Fixed | Amlodipine | T40/A5 | non-ACEI | 156 | 55.4(9.1) | 73.1 |
|  |  |  |  |  |  |  | T40 | non-ACEI | 158 | 55.5(10.2) | 77.8 |
| Yoshinaga, 2008 [8] | I | Mild-moderate essential HT | Out | 12w | Escalation | Enalapril | I50-100 | ACEI | 135 | 56.8(9.0) | 67.4 |
| Yoshinaga, 2008 [21] | I | Mild-moderate essential HT | Out | 8w | Escalation | Losartan | I100-200 | non-ACEI | 198 | 60(28-79)^a^ | 67.2 |
| E2233 | I | Mild-moderate essential HT | Out | 6w | Fixed | Placebo | I100 | non-ACEI | 39 | NA^b^ | 73.7 |

^a^ Median (min-max).

^b^ Data not available. The layer of 60-69 was 34.2% and the modal class.

HCTZ, Hydrochlorothiazide; L, Losartan; C, Candesartan; V, Valsartan; O, Olmesartan; T, Telmisartan; I, Irbesartan; H, Hydrochlorothiazide; A, Amlodipine; AZ, Azelnidipine.
