# Supplementary material for: Biased Safety Reporting in Blinded Randomized Clinical Trials: Meta-Analysis of Angiotensin Receptor Blocker Trials
Source: PLoS One. 2013 Sep 23;8(9):e75027. doi: 10.1371/journal.pone.0075027 (PMC3781030; doi:10.1371/journal.pone.0075027)
Supplement: Table S2 — Evaluation of risk of bias. (DOC) [file pone.0075027.s002.doc]

Table S2. Evaluation of risk of bias.

| ARB Name | Study | Random sequence generation | Allocation concealment | Blinding of participants and personnel | Blinding of outcome assessment | Incomplete outcome data | Selective reporting | Other sources of bias (possibility of unblinding due to adverse event, cough) | Other sources of bias (possibility of unblinding due to adverse event, headache) |
| --- | --- | --- | --- | --- | --- | --- | --- | --- | --- |
| Losartan | Yoshinaga et al, 1995 [3] | Low | Low | Low | Low | Low | Low | Unclear | Low |
|  | Sumita et al, 1995 [20] | Low | Unclear | Low | Low | Low | Low | Low | Low |
|  | No301 | Low | Low | Low | Low | Low | Low | Low | Low |
| Candesartan | Arakawa et al, 1998 [4] | Low | Low | Low | Low | Low | Low | Unclear | Low |
|  | CCT-002 | Low | Low | Low | Low | Low | Low | Low | Low |
|  | CCT-001 | Low | Low | Low | Low | Low | Low | Low | Low |
|  | CCT-001 | Low | Low | Low | Low | Low | Low | Low | Low |
| Valsartan | Yoshinaga et al, 1998 [5] | Low | Low | Low | Low | Low | Low | Unclear | Low |
|  | 1301 | Low | Low | Low | Low | Low | Low | Low | Low |
|  | 1303 | Low | Low | Low | Low | Low | Low | Low | Low |
|  | 1301 | Low | Low | Low | Low | Low | Low | Low | Low |

| ARB Name | Study | Random sequence generation | Allocation concealment | Blinding of participants and personnel | Blinding of outcome assessment | Incomplete outcome data | Selective reporting | Other sources of bias (possibility of unblinding due to adverse event, cough) | Other sources of bias (possibility of unblinding due to adverse event, headache) |
| --- | --- | --- | --- | --- | --- | --- | --- | --- | --- |
| Olmesartan | Arakawa et al, 2004 [6] | Low | Low | Low | Low | Low | Low | Unclear | Low |
|  | 5-34 | Low | Low | Low | Low | Low | Low | Low | Low |
|  | J301 | Low | Low | Low | Low | Low | Low | Low | Low |
|  | J201 | Low | Low | Low | Low | Low | Low | Low | Low |
| Telmisartan | Arakawa et al, 2002 [7] | Low | Low | Low | Low | Low | Low | Low | Low |
|  | 502.439 | Low | Low | Low | Low | Low | Low | Low | Low |
|  | 502.436 | Low | Low | Low | Low | Low | Low | Low | Low |
|  | 1235.13 | Low | Low | Low | Low | Low | Low | Low | Low |
|  | 1235.14 | Low | Low | Low | Low | Low | Low | Low | Low |
| Irbesartan | Yoshinaga, 2008 [8] | Low | Low | Low | Low | Low | Low | Unclear | Low |
|  | Yoshinaga, 2008 [21] | Low | Low | Low | Low | Low | Low | Low | Low |
|  | E2233 | Low | Low | Low | Low | Low | Low | Low | Low |
